# Author Correction: MYC-driven epigenetic reprogramming favors the onset of tumorigenesis by inducing a stem cell-like state

**DOI:** 10.1038/s41467-018-06480-y

**Published:** 2018-09-20

**Authors:** Vittoria Poli, Luca Fagnocchi, Alessandra Fasciani, Alessandro Cherubini, Stefania Mazzoleni, Sara Ferrillo, Annarita Miluzio, Gabriella Gaudioso, Valentina Vaira, Alice Turdo, Miriam Gaggianesi, Aurora Chinnici, Elisa Lipari, Silvio Bicciato, Silvano Bosari, Matilde Todaro, Alessio Zippo

**Affiliations:** 1 Laboratory of Chromatin Biology & Epigenetics, Center for Integrative Biology (CIBIO), University of Trento, 38123 Trento, Italy; 2 Fondazione Istituto Nazionale di Genetica Molecolare “Romeo ed Enrica Invernizzi”, Via F. Sforza 35, 20122 Milan, Italy; 3 Division of Pathology, Fondazione IRCCS Ca’ Granda Ospedale Maggiore Policlinico, Milan, 20122 Italy; 4 Department of Pathophysiology and Transplantation, University of Milan, Milan, 20122 Italy; 5 Department of Surgical, Oncological and Stomatological Sciences, University of Palermo, Palermo, 90127 Italy; 6 Center for Genome Research, University of Modena and Reggio Emilia, Modena, 41125 Italy; 7 DiBiMIS, University of Palermo, Palermo, 90127 Italy

Correction to: *Nature Communications*; 10.1038/s41467-018-03264-2; published online 9 March 2018

The original version of this Article contained an error in the spelling of the author Miriam Gaggianesi, which was incorrectly given as Miriam Giaggianesi. Furthermore, the affiliation details for Gabriella Gaudioso, Valentina Vaira and Silvano Bosari incorrectly omitted ‘Division of Pathology, Fondazione IRCCS Ca’ Granda Ospedale Maggiore Policlinico, Milan, 20122, Italy’. Finally, the affiliation details for Alice Turdo, Miriam Gaggianesi, Aurora Chinnici and Elisa Lipari were incorrectly given as ‘Dipartimento di Biotecnologie Mediche e Medicina Legale Sezione di Biochimica Medica, Facoltà di Medicina e Chirurgia, Policlinico “P.Giaccone”, Università di Palermo, Palermo, 90127, Italy’. The correct affiliation is ‘Department of Surgical, Oncological and Stomatological Sciences, University of Palermo, Palermo, 90127, Italy’. These errors have now been corrected in both the PDF and HTML versions of the Article.

