# Supplementary material for: MYC-driven epigenetic reprogramming favors the onset of tumorigenesis by inducing a stem cell-like state
Source: Nat Commun. 2018 Mar 9;9:1024. doi: 10.1038/s41467-018-03264-2 (PMC5844884; doi:10.1038/s41467-018-03264-2)
Supplement: Supplementary file 1 — Supplementary Information [file 41467_2018_3264_MOESM1_ESM.pdf]

Supplementary Information

MYC-driven epigenetic reprogramming favors the onset of tumorigensis by inducing a stem cell-like state

Poli et al.

Supplementary Figures

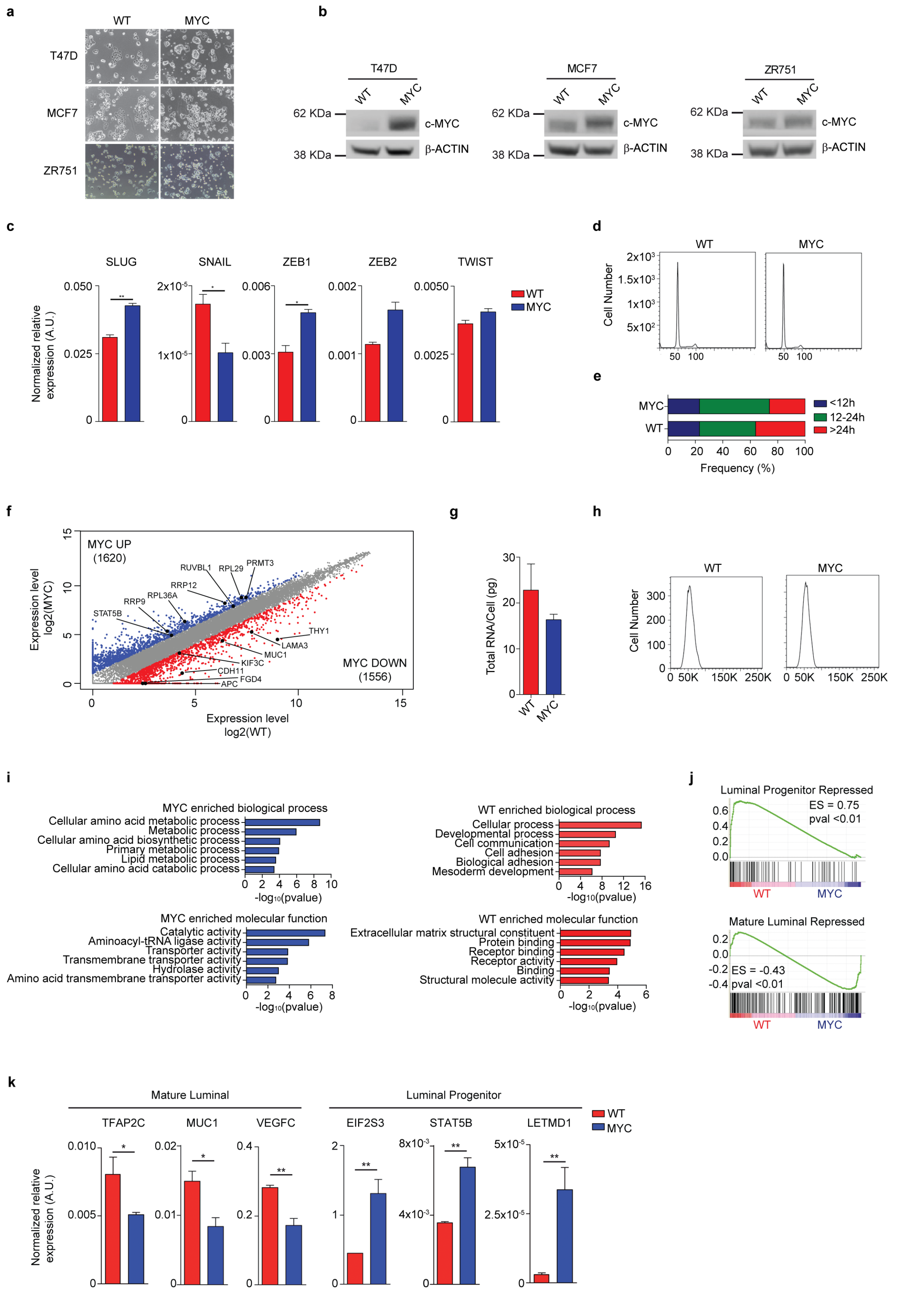

Poli_Supplementary Figure 1

Supplementary Figure 1.

(a) Phase contrast photographs showing the morphology of WT and MYC-overexpressing luminal breast cancer cell lines. Scale bar, 100 μm. **(b)** Western blot analysis of c-MYC in WT and MYC-overexpressing luminal breast cancer cell lines; β-ACTIN was used as loading control. **(c)** qRT-PCR analysis of EMT genes (SLUG, SNAIL, ZEB1, ZEB2 and TWIST) on IMEC WT and IMEC-MYC. Relative transcript levels are normalized on GAPDH. Data are means +/- SEM (n=3). (*P<0.05, **P<0.01; Student's *t*-test). **(d)** Cell cycle profile of IMEC WT and IMEC-MYC. **(e)** Frequency of cell divisions of IMEC WT and IMEC-MYC in the indicated time windows. **(f)** Scatterplot analysis of gene expression profile of IMEC WT and IMEC-MYC. Genes up- (blue) and down-regulated (red) in IMEC-MYC, with -2 > fold change > 2 respect to IMEC WT, are highlighted. Relevant genes are indicated, among up- and down-regulated genes in IMEC-MYC. (n=3). **(g)** Quantification of total RNA/cell in IMEC WT and -MYC. Data are means +/- SEM (n=3). **(h)** FACS analysis of cell size distribution (FSC) of IMEC WT and MYC. **(i)** Gene ontology analysis of differentially regulated genes between IMEC WT and IMEC-MYC, showing relative enriched biological processes and molecular functions (n=3). **(j)** Gene set enrichment analysis (GSEA) of mature luminal and luminal progenitor cell repressed gene signatures in IMEC WT versus IMEC-MYC (n=3). **(k)** qRT-PCR analysis of mature luminal (TFAP2C, MUC1 and VEGFC) and luminal progenitor (EIF2S3, STAT5B and LETMD1) markers on IMEC WT and IMEC-MYC. Relative transcript levels are normalized on spike-in RNAs. Data are means +/- SEM (n=3). (*P<0.05, **P<0.01; Student's *t*-test).

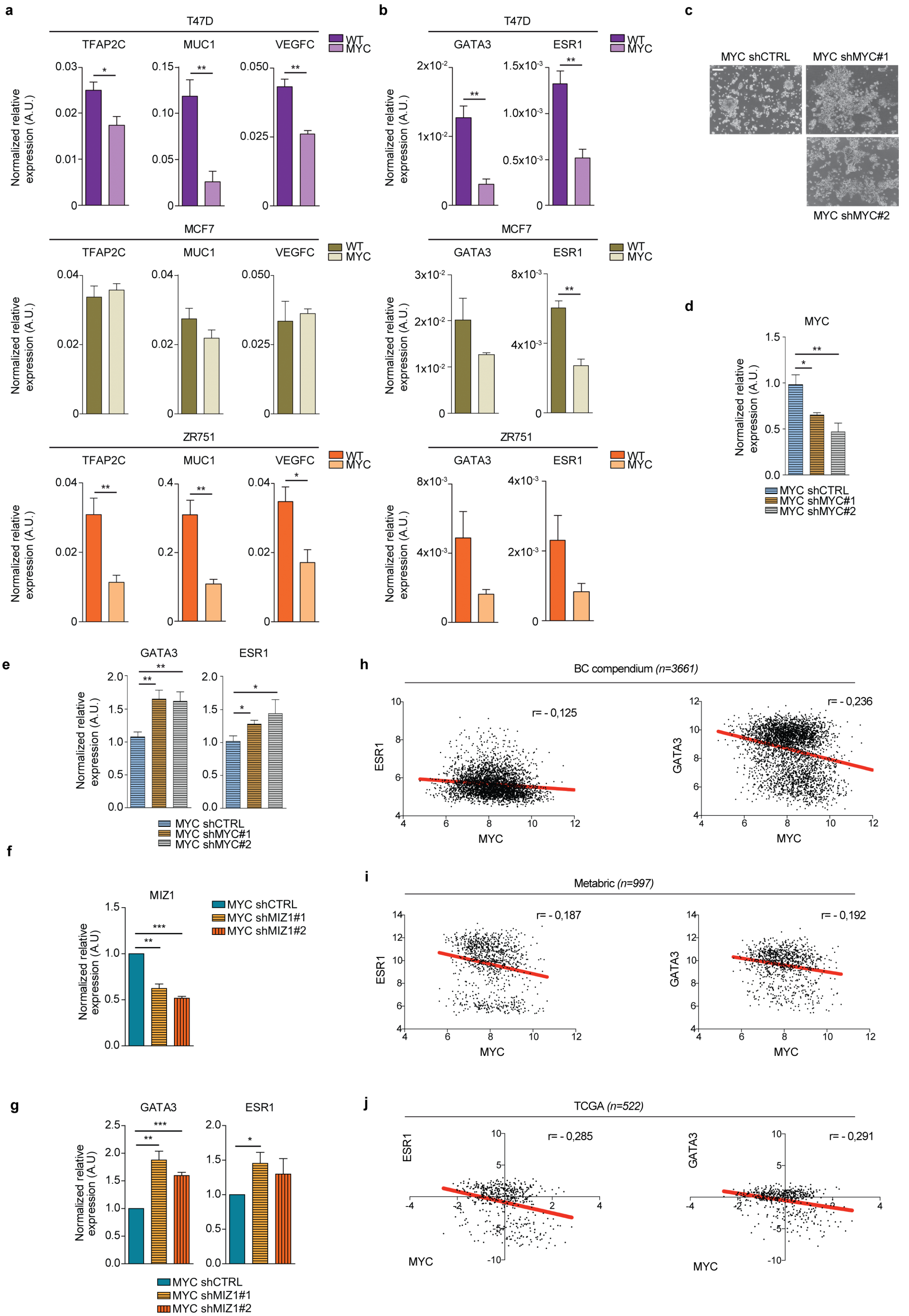

Poli_Supplementary Figure 2

Supplementary Figure 2.

(a) qRT-PCR analysis of mature luminal markers (TFAP2C, MUC1 and VEGFC) on WT and MYC-overexpressing luminal breast cancer cell lines. Relative transcript levels are normalized on spike-in RNAs. Data are means +/- SEM (n=3). (*P<0.05, **P<0.01; Student's *t*-test). **(b)** qRT-PCR analysis of mature luminal TFs (GATA3 and ESR1) on WT and MYC-overexpressing luminal breast cancer cell lines. Relative transcript levels are normalized on spike-in RNAs. Data are means +/- SEM (n=3). (*P<0.05, **P<0.01, ***P<0.001; Student's *t*-test). **(c)** Phase contrast images of confluent IMEC-MYC expressing IPTG-inducible shRNAs targeting control (Ctrl) and murine MYC transcript (shMYC #1, shMYC#2), in presence of 500 µM IPTG to down-regulate the expression level of the exogenous MYC. Scale bar, 200 µm. **(d)** qRT-PCR analysis of exogenous MYC on IMEC-MYC, expressing IPTG-inducible shRNAs targeting control (Ctrl) and murine MYC transcript (shMYC #1, shMYC #2), in presence of 500 µM IPTG. Relative transcript levels are normalized on GAPDH and un-induced sample values. Data are means +/- SEM (n=3). (*P<0.05, **P<0.01; Student's *t*-test). **(e)** qRT-PCR analysis of ESR1 and GATA3 on IMEC-MYC, expressing IPTG-inducible shRNAs targeting control (CTRL) and murine MYC transcript (shMYC #1, shMYC #2), in presence of 500 µM IPTG. Relative transcript levels are normalized on GAPDH and un-induced sample values. Data are means +/- SEM (n=3). (*P<0.05, **P<0.01; Student's *t*-test). **(f)** qRT-PCR analysis of MIZ1 on IMEC-MYC, expressing shRNAs targeting control (CTRL) and MIZ1 transcript (shMIZ1 #1, sh MIZ1 #2). Relative transcript levels are normalized on GAPDH. Data are means +/- SEM (n=3). (**P<0.01, ***P<0.001; Student's *t*-test). **(g)** qRT-PCR analysis of ESR1 and GATA3 on IMEC-MYC, expressing shRNAs targeting control (Ctrl) and MIZ1 transcript (shMIZ1 #1, sh MIZ1 #2). Relative transcript levels are normalized on GAPDH. Data are means +/- SEM (n=3). (*P<0.05, **P<0.01, ***P<0.001; Student's *t*-test). **(h-j)** Scatter plot (black dots) and linear regression (red line) of expression values indicating the negative correlation between MYC

and ESR1 (left panels) and GATA3 (right panels) gene expression levels in primary human breast cancers. Data from **(h)** the BC compendium (n = 3,661); **(i)** the METABRIC collection (n = 997); **(j)** the TCGA dataset (n = 522). Pearson r quantifies the linear dependence between the levels of the two genes (P-value < 0.0001).

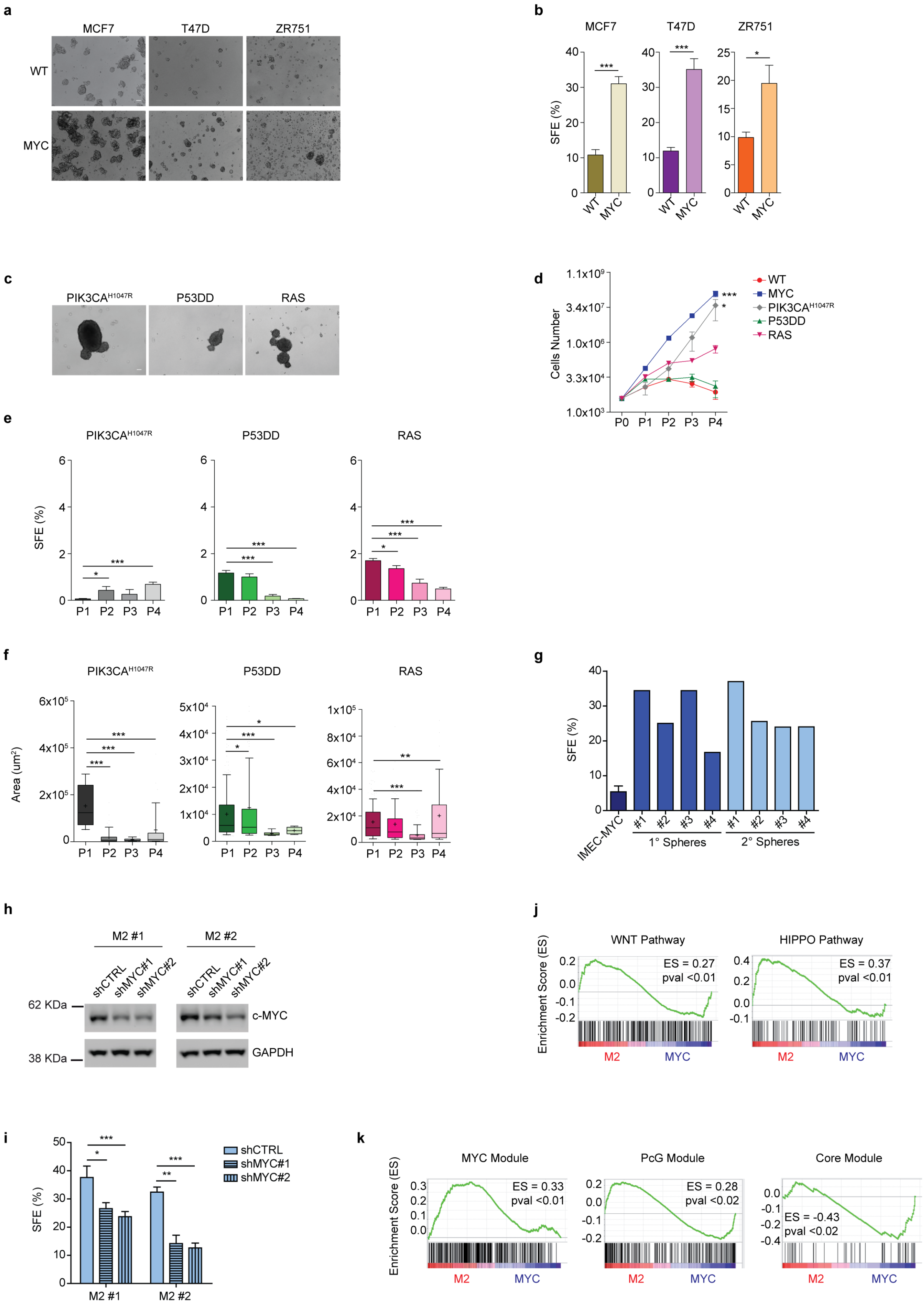

Poli_Supplementary Figure 3

Supplementary Figure 3.

(a) Phase contrast photographs showing WT and MYC-overexpressing luminal breast cancer cell lines cultured in low adhesion conditions. Scale bar, 100 μm. **(b)** Spheres formation efficiency (SFE) of WT and MYC-overexpressing luminal breast cancer cell lines cultured in low adhesion condition at passage 1 (*P<0.05, ***P<0.001; Student's t test). **(c)** Phase contrast photographs showing IMEC-PIK3CA^{H1047R}, -P53DD and -RAS cultured in low adhesion conditions. Scale bar, 100 μm. **(d)** Growth curve of IMEC WT, -MYC, -PIK3CA^{H1047R}, -P53DD and –RAS cultured in low adhesion conditions for 4 subsequent passages. Data are means +/- SEM (n=6). (*P<0.05, ***P<0.001; 2way ANOVA). **(e)** Spheres formation efficiency (SFE) of IMEC-PIK3CA^{H1047R}, -P53DD and -RAS cultured in low adhesion conditions at indicated passages (n=6). (*P<0.05, ***P<0.001, Student's *t*-test). **(f)** Area (μm^2) of mammospheres formed by IMEC-PIK3CA^{H1047R}, -P53DD and -RAS cultured in low adhesion conditions at indicated passages (n=6). Boxes encompass the 25th to 75th percentiles; whiskers extend to 10th and 90th percentiles; the central horizontal bar indicates median fold change, the black cross indicates the mean. (*P<0.05, **P<0.01, ***P<0.001; Student's *t*-test). **(g)** Spheres formation efficiency (SFE) of IMEC-MYC and individual clones of both primary and secondary mammospheres. **(h)** Western blot of c-MYC in two independent clones of secondary mammospheres expressing IPTG-inducible shRNAs targeting control (CTRL) and murine MYC transcript (shMYC #1, shMYC #2) and grown in presence of 500 μM IPTG to down-regulate the expression level of the exogenous MYC.; GAPDH was used as loading control. **(i)** Spheres formation efficiency (SFE) at single cell level of two independent clones of secondary mammospheres expressing the indicated shRNAs. **(j)** Gene set enrichment analysis (GSEA) of WNT and HIPPO pathway genes in IMEC-MYC versus M2 (n=3). **(k)** Gene set enrichment analysis (GSEA) showing the gene activity of the three embryonic stem cell (ESCs) modules (MYC module, PcG module and core module) in IMEC-MYC versus M2 (n=3).

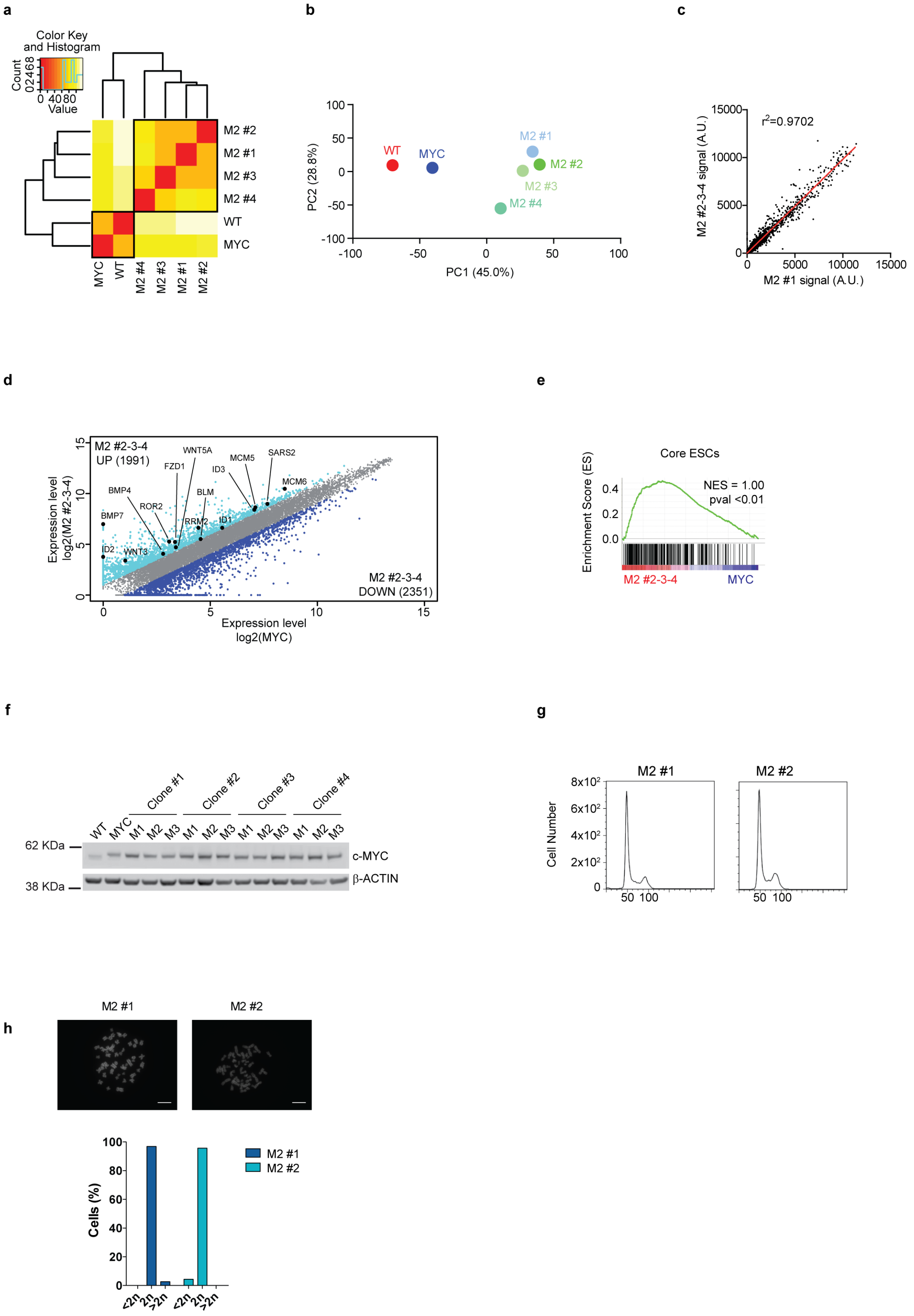

Poli_Supplementary Figure 4

Supplementary Figure 4

(a) Heatmap showing the correlation between the transcriptional profiles of IMEC WT, -MYC and the four independent clones of M2 mammospheres (M2#1-4) analyzed by microarray, measured by Euclidean distance. IMEC WT, MYC and M2#1 represent the average of three biological replicates. **(b)** Principal component analysis (PCA) of the transcriptional profiles of IMEC WT, -MYC and the four independent clones of M2 mammospheres (M2#1-4). PC = principal component. IMEC WT, MYC and M2#1 represent the average of three biological replicates. **(c)** Scatterplot of expression values from microarray experiments between M2#1 (biological triplicate) and averaged signals of other three independent mammosphere clones (M2#2-3-4). A.U. = arbitrary units. **(d)** Scatterplot of gene expression profile of IMEC-MYC and averaged signals of other three independent mammosphere clones (M2#2-3-4). Genes up- (cyano) and down-regulated (blue) in M2#2-3-4 respect to IMEC-MYC are highlighted. Relevant M2#2-3-4 up-regulated genes are indicated. **(e)** GSEA of the core embryonic stem cell (ESCs) gene module in IMEC-MYC versus M2#2-3-4. **(f)** Western blot analyses of MYC protein levels in IMEC-WT, -MYC and in four independent clones of primary (M1), secondary (M2) and tertiary (M3) mammospheres. β-ACTIN was used as loading control. **(g)** Cell cycle profile of two independent-derived clones of secondary (M2) mammospheres, maintained in culture for more than 30 passages. **(h)** Karyotype analyses in two independent clones of late passages (M2) mammospheres, showing for each sample the normal number of chromosomes of humane diploid nuclei (46 chromosomes). Karyotype analyses were performed on more than 40 individual nuclei for each clone, resulting in 98% of euploid cells (2n). Chromosomes are fluorescently stained with DAPI; scale bar 10 μm.

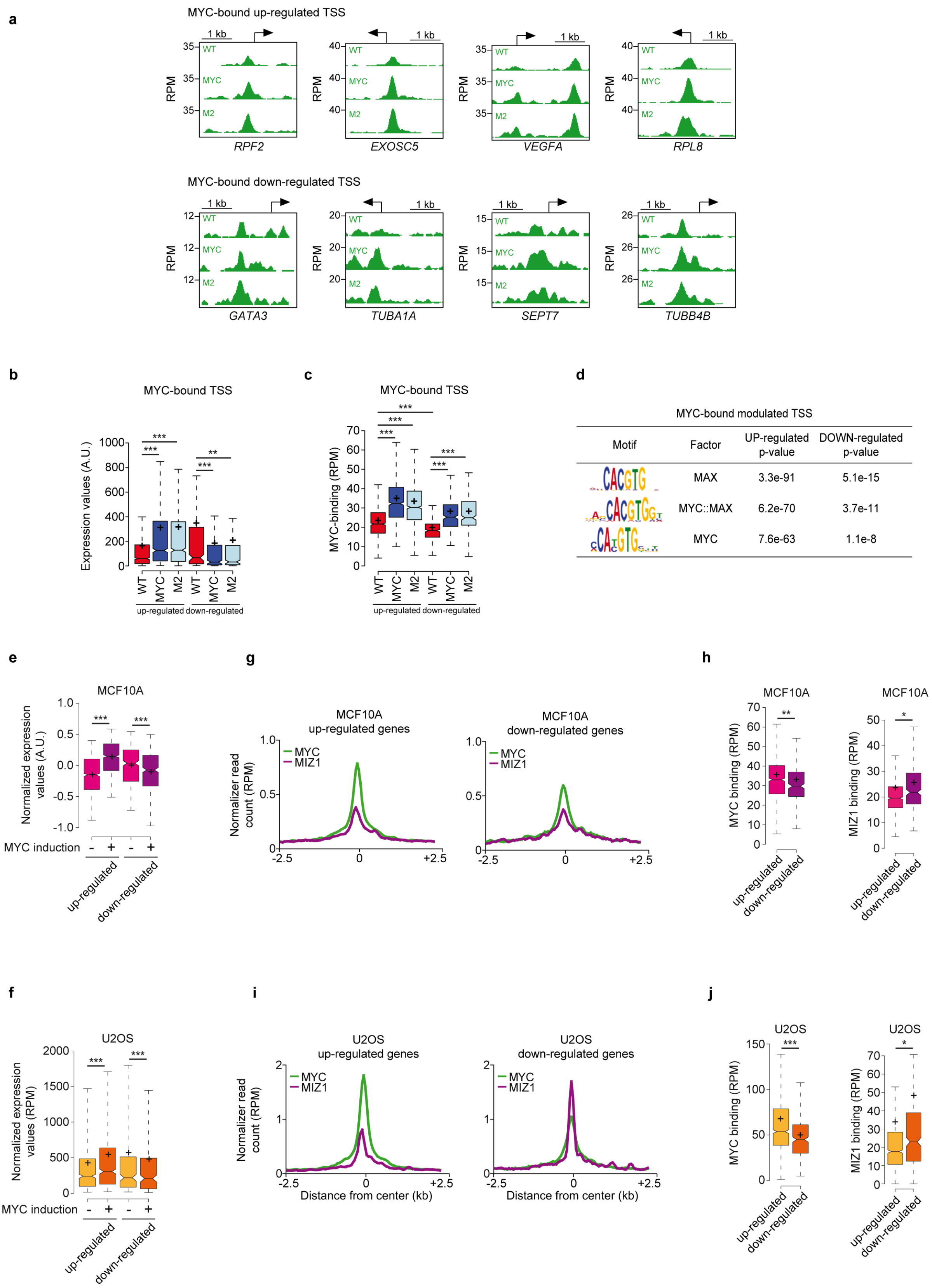

Poli_Supplementary Figure 5

Supplementary Figure 5

(a) Genomic snapshots showing MYC binding at the transcriptional start sites of up- (upper panels) and down-regulated (lower panels) MYC targets, in IMEC WT, MYC and mammospheres. 1 kb scale bars and black arrows indicating gene orientation are reported on each snapshot. RPM = reads per million. **(b-c)** Notched boxplot showing the distribution of expression values **(b)** and MYC binding **(c)** on up- and down-regulated genes bound by MYC at their TSS in IMEC WT, IMEC-MYC and M2. The horizontal black lines and black crosses indicate the median and the average of each distribution, respectively. The boxes extend from the 1st to 3rd quartile and the Tukey method was used to plot whiskers (**P<0.01, ***P<0.001; Student's t-test). **(d)** Table depicting MYC and MAX binding sites enrichment at the TSS of up- and down-regulated genes MYC target genes. **(e)** Notched boxplot showing the normalized expression levels of up- and down-regulated genes in MCF10A cell line, upon MYC induction. The horizontal black lines and black crosses indicate the median and the average of each distribution, respectively (***P<0.001; Student's t-test). A.U. = arbitrary units. **(f)** Tag density plots of MYC and MIZ1 normalized ChIP-seq signals in MCF10A, centered at TSS of up- (left) and down-regulated (right) genes, upon MYC induction (***P<0.001; Student's t-test). RPM = reads per million. **(g)** Notched boxplot showing MYC (left) and MIZ1 (right) binding distribution at the TSS of up- and down-regulated genes upon MYC induction in MCF10A cell line. The horizontal black lines and black crosses indicate the median and the average of each distribution, respectively (*P<0.05, **P<0.01; Student's t-test). RPM = reads per million. **(h)** Notched boxplot showing the normalized expression levels of up- and down-regulated genes in U2OS cell line, upon MYC induction. The horizontal black lines and black crosses indicate the median and the average of each distribution, respectively. RPM = reads per million. **(i)** Tag density plots of MYC and MIZ1 normalized ChIP-seq signals in U2OS, centered at TSS of up- (left) and down-regulated (right) genes, upon MYC induction. RPM = reads per million. **(j)**

Notched boxplot showing MYC (left) and MIZ-1 (right) binding distribution at the TSS of up- and down-regulated genes upon MYC induction in U2OS cell line. The horizontal black lines and black crosses indicate the median and the average of each distribution, respectively (*P<0.05, ***P<0.001; Student's t-test). RPM = reads per million.

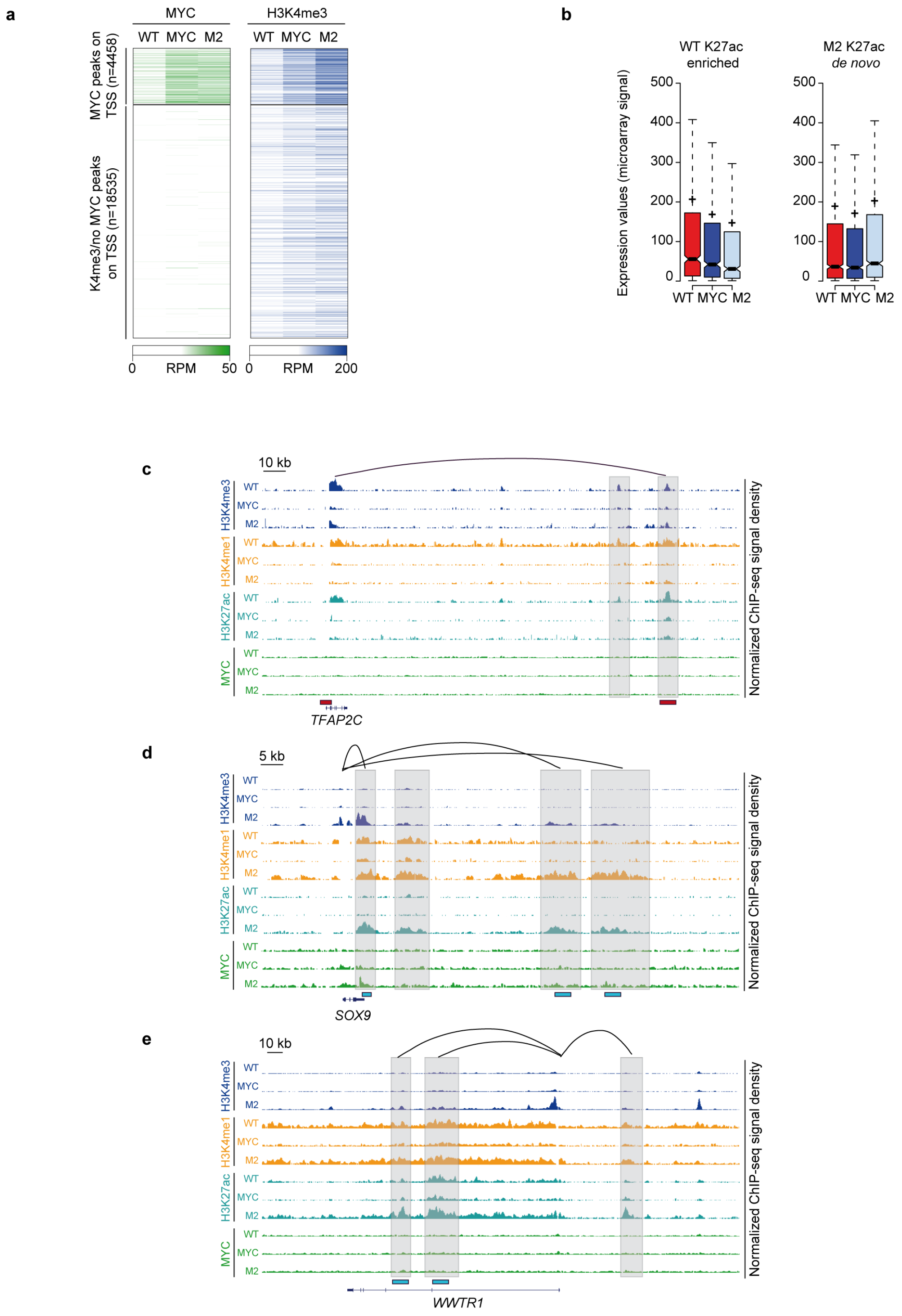

Poli_Supplementary Figure 6

Supplementary Figure 6.

(a) Heatmap showing the dynamic behavior of MYC and H3K4me3 normalized ChIP-seq signals on active promoters (TSS) in IMEC WT, IMEC-MYC and M2. RPM = reads per million. **(b)** Notched boxplots showing the distribution of expression values of genes associated to enhancers enriched for H3K27ac in IMEC WT, and M2, as indicated. The horizontal black lines and black crosses indicate the median and the average of each distribution, respectively. The boxes extend from the 1st to 3rd quartile and the Tukey method was used to plot whiskers. **(c-e)** Genomic snapshots showing the epigenetic landscape and MYC binding at relevant genes associated to modulated enhancers. TFAP2C **(c)** is shown as representative example of gene associated to enhancer down-regulation between IMEC WT and IMEC-MYC/M2, while SOX9 **(d)** and WWTR1 **(e)** represent examples of genes related to activated *de novo* enhancers in M2, either bound or not by MYC, respectively. Light grey vertical bars indicate enhancer regions. Horizontal red bars indicate multiple binding sites for GATA3/ESR1/FOXA1/ZNF1, while cyano horizontal bars indicates DNase hypersensitive sites which posses multiple unspecific transcription factor binding sites (data from ENCODE). The physical interaction of each TSS with enhancer regions is indicated by the curved lines as indicated by previously published ChIA-PET data. The x axis corresponds to genomic location, while the y axis corresponds to ChIP-seq signal density normalized to sequencing depth. Scale bars are reported.

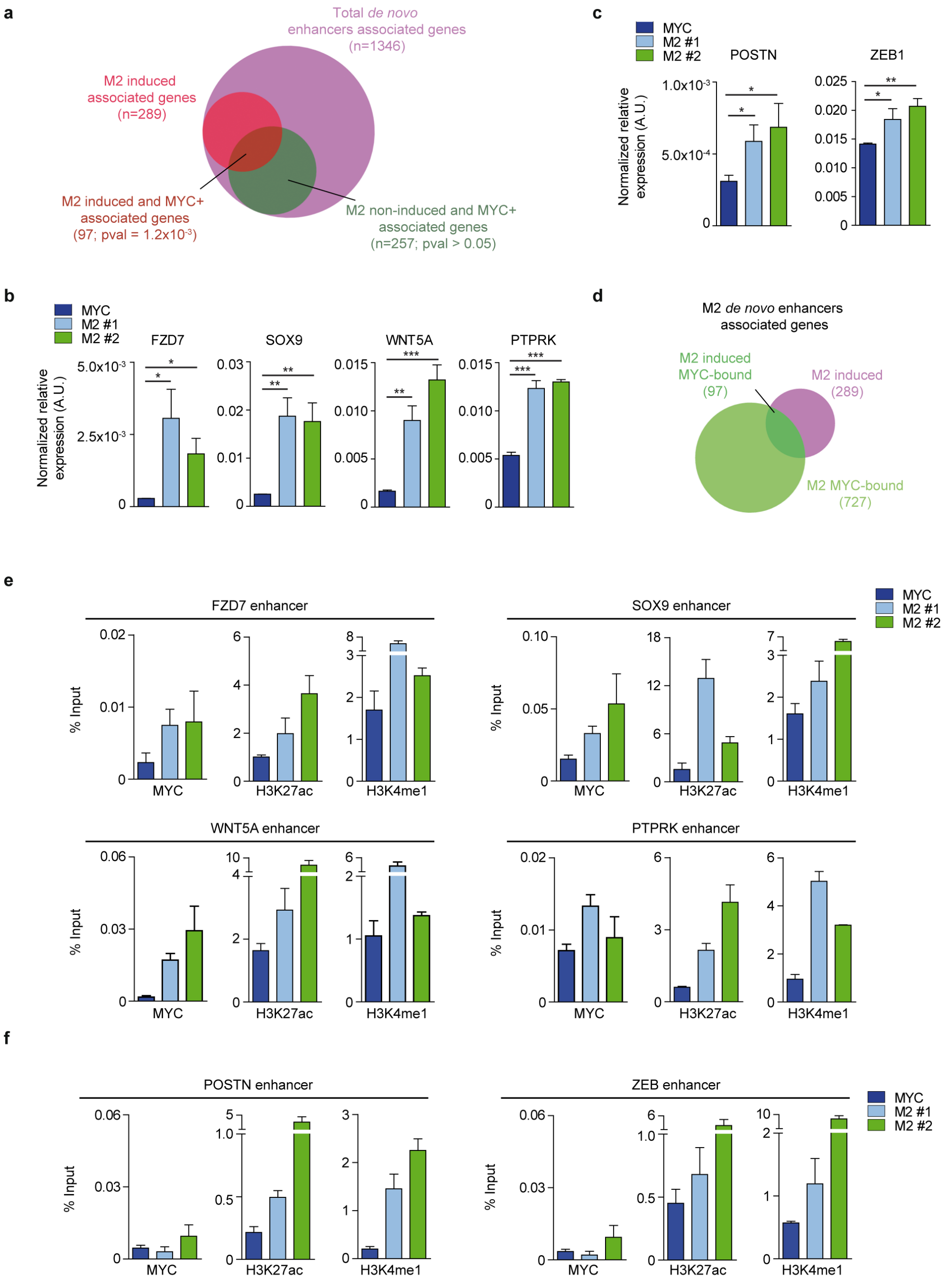

Poli_Supplementary Figure 7

Supplementary Figure 7.

(a) Venn diagrams showing the overlap between M2 *de novo* enhancers associated genes, which are either at least two-fold transcriptionally induced in the comparison IMEC-MYC versus M2 (red circle) or not induced but enriched for MYC binding in mammospheres (green circle). The total number of genes belonging to each group is reported. The probability of transcriptionally induced genes marked by increased MYC binding at their enhancers is associated to a pval = $1.2x10^{-3}$, as calculated by an hypergeometric test. **(b)** qRT-PCR analysis of genes associated to the MYC-bound M2 *de novo* enhancers in IMEC-MYC and M2 clones (M2 #1 and M2 #2). Relative transcript levels are normalized on spike-in RNAs. Data are means +/- SEM (n=3). (*P<0.05, **P<0.01, ***P<0.001; Student's *t*-test). **(c)** qRT-PCR analysis of genes associated to M2 *de novo* enhancers, on which MYC is not bound, in IMEC-MYC and M2 clones. Relative transcript levels are normalized on spike-in RNAs. Data are means +/- SEM (n=3). (*P<0.05 , **P<0.01; Student's *t*-test). **(d)** Venn diagram showing the overlap between M2 *de novo* enhancers-associated genes, which are either transcriptionally induced or enriched for MYC binding, with at least a two-fold change in M2 with respect to IMEC-MYC. **(e)** ChIP-qPCRs assessing MYC binding and H3K27ac and H3K4me1 deposition at the MYC-bound M2 *de novo* enhancers in IMEC-MYC and M2 clones. Data are means +/- SEM (n=3). **(f)** ChIP-qPCRs assessing MYC binding and H3K27ac and H3K4me1 deposition at the M2 *de novo* enhancers, on which MYC is not bound, in IMEC-MYC and M2 clones. Data are means +/- SEM (n=3).

a

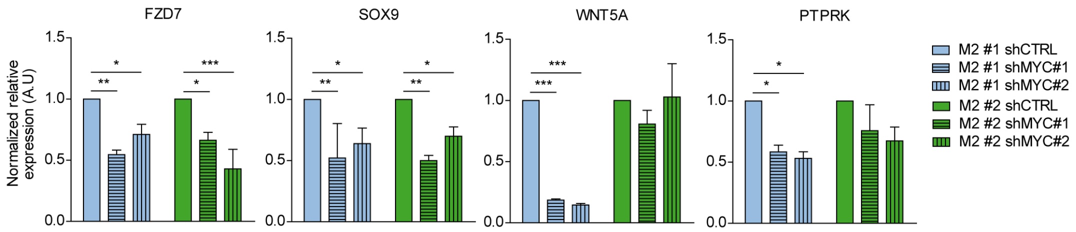

b

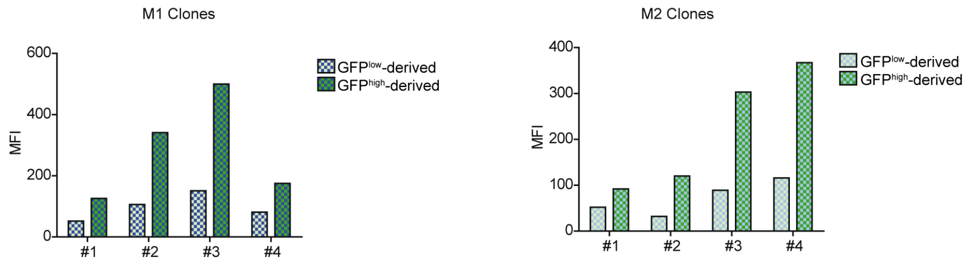

Poli_Supplementary Figure 8

Supplementary Figure 8

(a) qRT-PCR of *de novo* enhancer-related genes on secondary mammospheres (M2 #1 and M2 #2) expressing IPTG-inducible shRNAs targeting control (Ctrl) and murine MYC transcript (shMYC #1, shMYC #2), in presence of 500 μM IPTG. Relative transcript levels are normalized on spike-in RNAs and un-induced sample values. Data are means +/- SEM (n=3). (*P<0.05, **P<0.01, ***P<0.001; Student's *t*-test). **(b)** On the left, single cell spheres formation efficiency (SFE) of four independent clones of GFP^{high} and GFP^{low} cells sorted from IMEC-MYC-7TGP, which gave rise to M1. MFI of GFP^{high}- and GFP^{low}-derived M1 is reported. On the right, single cell SFE of four independent clones of GFP^{high} and GFP^{low} cells sorted from GFP^{high}-derived M1, which gave rise to M2. MFI of GFP^{high}- and GFP^{low}-derived M2 is reported.

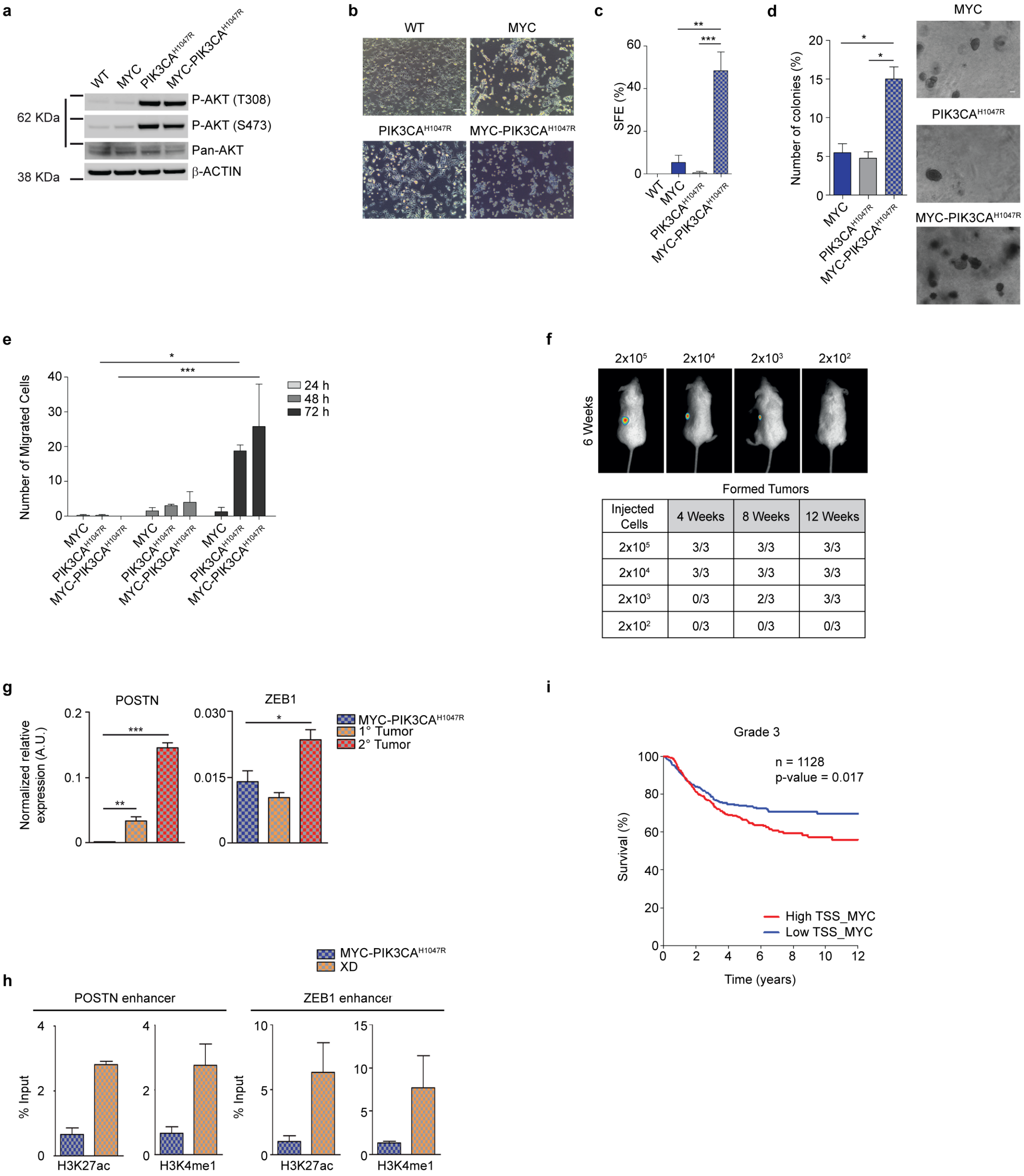

Injected Cells	4 Weeks	8 Weeks	12 Weeks
2x10^5	3/3	3/3	3/3
2x10^4	3/3	3/3	3/3
2x10^3	0/3	2/3	3/3
2x10^2	0/3	0/3	0/3

Poli_Supplementary Figure 9

Supplementary Figure 9.

(a) Western blot analysis of Pan-AKT, Phospho-AKT (S473) and Phospho-AKT (T308) in IMEC WT, -MYC, -PIK3CA^{H1047R} and -MYC-PIK3CA^{H1047R}; β-ACTIN was used as loading control. **(b)** Phase contrast photographs showing the morphology of IMEC WT, -MYC, -PIK3CA^{H1047R} and -MYC-PIK3CA^{H1047R}. Scale bar, 100 μm. **(c)** Single cell spheres formation efficiency (SFE) of IMEC WT, -MYC, -PIK3CA^{H1047R} and -MYC-PIK3CA^{H1047R}. Data are means +/- SEM (n=3). (**P<0.01, ***P<0.001; Student's t test). **(d)** On the left, soft-agar colony forming assay on IMEC-MYC, -PIK3CA^{H1047R} and -MYC-PIK3CA^{H1047R} cells. Data are means +/- SEM (n=3). (*P<0.05; Student's t test). On the right, phase contrast photographs showing colonies formed by IMEC-MYC, -PIK3CA^{H1047R} and -MYC-PIK3CA^{H1047R} in soft-agar. Scale bar, 100 μm. **(e)** Invasion assay of IMEC-MYC, -PIK3CA^{H1047R} and -MYC-PIK3CA^{H1047R}. Data are means +/- SEM (n=4). (*P<0.05, ***P<0.001; 2way ANOVA). **(f)** In the upper panel, i*n vivo* imaging of NOD/SCID mice 6 weeks after injection in the sub-renal capsule of $2x10^5$, $2x10^4$, $2x10^3$ and $2x10^2$ IMEC-MYC-PIK3CA^{H1047R} (n=3). In the lower panel, table depicting the number of formed tumors per number of injections in the 4 dilutions, at the indicated time points. **(g)** qRT-PCR analysis of genes associated to the M2 *de novo* enhancers on which MYC is not bound, in IMEC-MYC-PIK3CA^{H1047R} and primary and secondary xenograft tumor cells. Relative transcript levels are normalized on spike-in RNAs. Data are means +/- SEM (n=3). (*P<0.05, **P<0.01, ***P<0.001; Student's *t*-test). **(h)** ChIP-qPCRs assessing H3K27ac and H3K4me1 deposition at the M2 *de novo* enhancers on which MYC is not bound, in IMEC-MYC-PIK3CA^{H1047R} and xenograft-derived (XD) cells. Data are means +/- SEM (n=3). **(i)** Kaplan–Meier analysis representing the probability of metastasis-free survival in 1128 grade 3 (G3) breast cancer patients from the meta-data set stratified according to high or low MYC direct target signature score. The log-rank test P-value reflects the significance of the association between high levels of the MYC direct target signature score and shorter survival.

Figure 1a

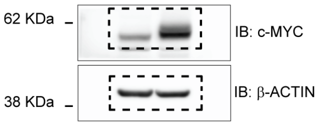

Figure S1b

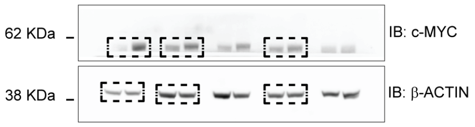

Figure S3h

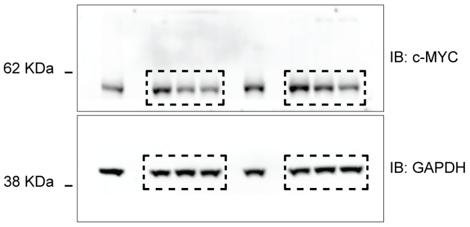

Figure S4f

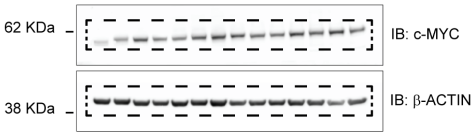

Figure S9a

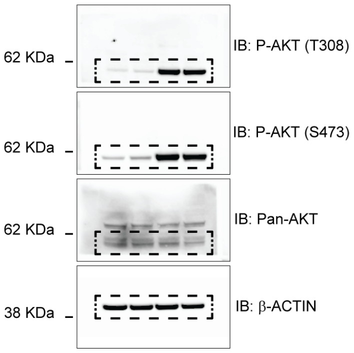

Poli_Supplementary Figure 10

Supplementary Figure 10 Uncropped WB images

Supplementary Table S1. List of *de novo* enhancer-associated genes, up-regulated in M2 respect to IMEC-MYC.

NO MYC enrichment in M2 with respect to HMEC-MYC (n=192)						MYC enrichment in M2 with respect to HMEC-MYC (n=97)		
SPP1	ZEB1	FRMD3	PRDM8	MGC27382	SELT	ACTL8	KIAA0513	SFRP2
SPARCL1	RERGL	ETNK1	PLCB1	POLR2B	TUBA1A	ADCY1	LAMA2	SH3GL3
HS3ST2	ARHGAP20	MANSC1	FARSB	LRP8	C5orf22	ADH1C	LPAR1	SORT1
LAMA4	AMPH	S1PR3	FOS	AGR3	METTL14	ARHGAP24	LRIG1	SOX9
PRRX2	LIFR	FZD1	ITPKB	BLM	REPS2	ARHGEF3	LUM	SPC24
COL6A2	ACER3	FZD4	FAM184A	SH3BP5	NEK6	ASB9	MAFB	SPRED1
HEY2	RHOJ	DDAH1	GYPC	CTDSPL2	LOC286297	B3GALNT1	MIR1265	SPRY2
MSMP	GLDC	PDGFD	PIK3R4	ARX	PBX1	BMF	MIR708	ST8SIA1
PTCHD1	PDK3	ADD3	UBASH3B	CCDC80	PITPNC1	BNC2	MITF	STRA6
RCAN2	WWTR1	LDB2	SPRY1	ERMP1	SCARNA23	C7	NEBL	SULT1E1
EYA1	WIPF1	PEG10	TMEM135	PPP1R12B	SLC20A2	CAMK1D	NPTX2	SYNPO2
FAM107A	POSTN	SLC12A2	KLRC2	SMARCA1	JAK2	CCDC102B	NR2F2	TBC1D16
COL6A1	LMNB1	EPHX1	PCDH18	CCDC88A		CDH11	NRP2	TCF4
FOXC1	TRIM62	STARD8	CHSY3	BUB1B		CGNL1	OLFM2	TDO2
GLI2	PKDCC	LOC340113	TBX3	TSHZ1		CLEC3B	PDGFRA	TFPI
COL6A3	TCF7L1	ARHGAP28	DHX9	C1orf21		CMTM8	PDGFRL	TIAM2
ANGPT1	USP18	SHISA2	DOCK11	BAIAP2L2		COL14A1	PI15	UACA
TMOD1	EPB41L4B	CABLES1	CNN3	RBM20		COL24A1	PKNOX2	WBP11P1
OGN	GPR150	ATP8B1	ART4	FAM105A		COL3A1	PLA2G4A	WNT5A
IGFBP5	MRAP2	ARHGAP18	FRMD5	HHEX		CSGALNACT1	PNMA2	WSCD1
CA4	MGP	GMPR	PHACTR2	MTMR2		CTGF	POP1	WWOX
SOBP	IL6R	GPR37	GALNT4	IL17D		CYTH3	PPM1H	ZNF280D
VAV3	PRUNE2	ARHGAP6	EXO1	TRPS1		DTNB	PREX1	ZNF521
CORO2B	LOC283585	TRIM67	LOC730101	MEGF10		DUSP6	PRMT6	ZNF592
VIPR1	RASGRP3	ITGA10	TOB1	RNF24		EDNRA	PTGFR	ZP1
CH25H	DENND2A	ID3	ATP11C	KIAA1958		ELOVL6	PTPRK	
SEPP1	TGFB3	DUT	KITLG	YES1		ENPP2	RAMP3	
TNFRSF11B	CA6	HELLS	SPRED2	CDC14C		ENSA	RDH10	
SIRPG	APCDD1L	IPMK	CHD1L	MARCKS		FAR2	RGL1	
PLAC9	RAB3IL1	ISM1	CAPN2	FAT3		FARP1	RGMA	
IGDCC4	JAM2	AGR2	CYB5B	COMMD9		FOXA1	ROR1	
FAM174B	SLC7A14	CHST2	GLYATL1	HABP4		FZD7	RORA	
EBF1	ZNF8	ASTN2	PDZRN3	RGN		FZD8	RSU1	
SNTB1	CLDN23	ZNF680	BICC1	MRPL13		GAS1	RTF1	
HSD17B7P2	ATP9A	ANTXR2	MCOLN2	TOP1		GRK5	SERBP1	
C6	SRPX2	MCM5	ZNF706	ARHGDIB		KHDRBS3	SESN3	

Supplementary Table S2. Original breast cancer datasets.

Study	Affymetrix platform	Samples	Data source	References
Stockholm	HG-U133A	159	GSE1456	Pawitan et al., 2005
EMC-286	HG-U133A	286	GSE2034	Wang et al., 2005
EMC-58	HG-U133A	58	GSE5327	Minn et al., 2007
MSK	HG-U133A	82	GSE2603	Minn et al., 2005
Uppsala-Miller	HG-U133A	236	GSE3494	Miller et al., 2005
Ivshina-Miller	HG-U133A	249	GSE4922	Ivshina et al., 2006
Loi	HG-U133A<br>HG-U133 Plus 2.0	414	GSE6532	Loi et al., 2007; Loi et al., 2008; Loi et al., 2010
Sotiriou	HG-U133A	187	GSE2990	Sotiriou et al., 2006
Tamoxifen	HG-U133 Plus 2.0	77	GSE9195	Loi et al., 2008;<br>Loi et al., 2010
Desmedt	HG-U133A	198	GSE7390	Desmedt et al., 2007
Schmidt	HG-U133A	200	GSE11121	Schmidt et al., 2008
Veridex	HG-U133A	136	GSE12093	Zhang et al., 2009
Chin	HG-U133AAofAV2	129	E-TABM-158	Merritt et al., 2008
Zhou	HG-U133AAofAV2	54	GSE7378	Zhou T et al., 2007;<br>Yau et al., 2008
TOP trial	HG-U133 Plus2.0	120	GSE16446	Desmedt et al., 2011; Li et al., 2010; Juul et al., 2010
GSE19615	HG-U133 Plus2.0	115	GSE19615	Li et al., 2010
IPC	HG-U133 Plus2.0	266	GSE21653	Sabatier et al., 2011
KFSYSCC	HG-U133 Plus2.0	327	GSE20685	Kao et al., 2011
GSE31519	HG-U133 Plus2.0	67	GSE31519	Rody et al., 2011; Karn et al., 2011; Karn et al., 2012
GSE22093	HG-U133A	103	GSE22093	Iwamoto et al., 2011
Hatzis	HG-U133A	508	GSE25066	Hatzis et al., 2011
GSE23988	HG-U133A	61	GSE23988	Iwamoto et al., 2011
GSE20271	HG-U133A	178	GSE20271	Tabchy et al., 2010
GSE20194	HG-U133A	230	GSE20194	Popovici et al., 2010; Shi et al., 2010
GSE32646	HG-U133 Plus2.0	115	GSE32646	Miyake et al., 2012
GSE18728	HG-U133 Plus2.0	24	GSE18728	Lin et al., 2010
GSE19697	HG-U133 Plus2.0	61	GSE19697	Korde et al., 2010

Supplementary Table S3: Independent cohorts comprised in the breast cancer meta-dataset.

Cohort	Affymetrix platform	Samples	Data source	References
KI_Stockholm	HG-U133A	159	GSE1456	Pawitan et al., 2005
EMC-344	HG-U133A	344	GSE2034<br>GSE5327	Wang et al., 2005;<br>Minn et al., 2007
MSKCC	HG-U133A	82	GSE2603	Minn et al., 2005
KI_Uppsala	HG-U133A	253	GSE3494<br>GSE4922<br>GSE6532	Loi et al, 2008;<br>Ivshina et al, 2006;<br>Miller et al, 2005
OXF	HG-U133A	178	GSE6532	Ivshina et al., 2006
GUY	HG-U133 Plus2.0	164	GSE6532<br>GSE9195	Loi et al., 2008;<br>Loi et al., 2010
TransBIG	HG-U133A	198	GSE7390	Desmedt et al., 2007
Mainz	HG-U133A	200	GSE11121	Schmidt et al., 2008
Veridex	HG-U133A	136	GSE12093	Zhang et al., 2009;<br>Loi et al., 2007;
UCSF	HG-U133AAofAV2	166	E-TABM-158<br>GSE7378	Merritt et al., 2008;<br>Zhou et al., 2007;<br>Yau et al., 2008
IJB_TOP	HG-U133 Plus2.0	114	GSE16446	Desmedt et al., 2011;<br>Li et al., 2010; Juul et al., 2010
US_NCI	HG-U133 Plus2.0	115	GSE19615	Li et al., 2010
CRCM	HG-U133 Plus2.0	252	GSE21653	Sabatier et al., 2011
KOOF	HG-U133 Plus2.0	327	GSE20685	Kao et al., 2011
Goethe	HG-U133A	64	GSE31519	Rody et al., 2011;<br>Karn et al., 2011;
MDACC_IGR	HG-U133A	61	GSE22093	Iwamoto et al., 2011;
I-SPY-1	HG-U133A	83	GSE25066	Hatzis et al., 2012
LBJ_INEN_GEICAM	HG-U133A	58	GSE25066	Hatzis et al., 2012
MDACC_GSE25066	HG-U133A	313	GSE25066<br>GSE20194	Hatzis et al., 2011;<br>Popovici et al., 2010;<br>Shi et al., 2010
USO-02103	HG-U133A	95	GSE23988	Iwamoto et al., 2011;<br>Hatzis et al., 2012
MDACC_GSE20271	HG-U133A	100	GSE20271	Tabchy et al., 2010
MDACC MAQC-II	HG-U133A	39	GSE20194	Popovici at al., 2010;<br>Shi et al., 2010
Osaka	HG-U133 Plus2.0	115	GSE32646	Miyake et al., 2012
UW	HG-U133 Plus2.0	21	GSE18728	Lin et al., 2010
St. Louis	HG-U133 Plus2.0	24	GSE19697	Korde et al., 2010

Supplementary Table S4: List of primers used for qRT-PCR and ChIP qPCR.

ID	qRT-PCR primers Sequence	Amplicon[1]
R29	CCGGTCCAGCACAGAAGGCA	GATA3_F
R30	GGGGCCGGTTCTGTCCGTTC	GATA3_R
Y73	CCAAATATCAGCACAGCACTTC	ESR1_F
Y74	AGGGCAGAAGGCTCAGAAAC	ESR1_R
AB21	GCCAGCTTGTGCCTAATAGAA	FZD7_F
AB22	AGCCGGGAGAAACTCACAG	FZD7_R
AB23	GTACCCGCACTTGCACAAC	SOX9_F
AB24	TCTCGCTCTCGTTCAGAAGTC	SOX9_R
AB29	ATAAGAGCCAGCACGGTCAA	PTPRK_F
AB30	CATAGTCAGGTAAAGTTGGAGCTG	PTPRK_R
AB31	ATTGTACTGCAGGTGTACCTTAAAAC	WNT5A_F
AB32	CCCCCTTATAAATGCAACTGTTC	WNT5A_R
B91	AGGCACTTACTTCCCTGCAA	LRP6_F
B92	CAAATTCCATAGTGTAATGTGATCG	LRP6_R
D29	CCGGCCCATCTACCCGTGTC	SFRP1_F
D30	ACCGTTGTGCCTTGGGGCTT	SFRP1_R
D33	TGTGCTTCGTGGGGCTTAACAA	FZD1_F
D34	AGCAGAAAGGACGTGCCGATAA	FZD1_R
XX01	CAGGCAGATGAAGCAGGATG	ZEB1_F
XX02	CACACCAGAAGCCAGTGGTC	ZEB1_R
AB33	CGAAGAGGACTGCGAGGA	TFAP2C_F
AB34	GGGGCTGTAGAGGTGCTG	TFAP2C_R
F67	CCTGCCTGAATCTGTTCTGC	MUC1_F
F68	CATGACCAGAACCCGTAACA	MUC1_R
Q54	TGCCAGCAACACTACCACAG	VEGFC_F
Q55	GTGATTATTCCACATGTAATTGGTG	VEGFC_R
T13	CTACCTGGTCTTCTTGCTAATGT	LETMD1_F
T14	AGGGATGACCTTTTCTAAATAACT	LETMD1_R
Q68	GGATGCAGCTCTTCTGTTGA	EIF2S3_F
Q69	TCTATAGCAGCCAGGTGTTCC	EIF2S3_R
S03	AGATGCTGGCCGAGGTCAAC	STAT5A/B_F
S04	AGACTTGGCCTGCTGCTCAC	STAT5A/B_R
AB13	TCAGTGCAGTCTTCTGAACCA	ZEB1_F
AB14	GAGGCTGATCATTGTTCTTGG	ZEB1_R
AB17	CACCAAGGTCACCAAATTCAT	POSTN_F
AB18	TTCCTCACGGGTGTGTCTC	POSTN_R
XX03	GCGCTTGACATCACTGAAGG	ZEB2_F
XX04	TGCTAACCCAAGGAGCAGGT	ZEB2_R
XX05	TCCTCTACCAGGTCCTCCA	TWIST_F
XX06	GAGACCTAGATGTCATTGTTTCC	TWIST_R
B79	CAGGCGTGCAAATCTGTCT	DKK1_F
B80	AATGATTTTGATCAGAAGACACACATA	DKK1_R
B87	CTCTGCTTCGTGTCCACCTT	FZD8_F
B88	GAAGCGCTCCATGTCGAT	FZD8_R

ID	qRT-PCR primers Sequence	Amplicon[1]
XX07	GCGAGCTGCAGGACTCTAA	SNAIL_F
XX08	GACAGAGTCCCAGATGAGC	SNAIL_R
XX09	AGATGCATATTCGGACCCAC	SLUG_F
XX10	CCTCATGTTTGTGCAGGAGA	SLUG_R

ID	ChIP qPCR primers Sequence	Amplicon[2]
T19	AGGGCTGGTTTCCTTGACTG	GATA3_pr_F
T20	CCCGGAGCCCTACTTACTC	GATA3_pr_R
G61	CTGGGCTCACTCACACATCT	ESR1_en_F
G62	AGGCATAGAGCAGAGTCACC	ESR1_en_R
AA67	CAGCAGCATTGTCTTTAAGCA	FZD7_en_F
AA68	ACCTAACATCGGGGAGTGTT	FZD7_en_R
AA69	AGATGCCTGCTCGCTCTG	SOX9_en_F
AA70	TGTCTTGAAGGTTAACTGCTGG	SOX9_en_R
AA77	AGCAGGACAAAGAGAGAGCA	PTPRK_en_F
AA78	CTCATGGGTGGAATTGGTGC	PTPRK_en_R
AB1	CACTGAGCCTCACCATCTCT	WNT5A_en_F
AB2	GTGGGGAGAGGAGAGGAAAC	WNT5A_en_R
AA57	TGAAGACTCAGACAGCCTCC	ZEB1_en_F
AA58	AACACCTCCATGCCTCTCTG	ZEB1_en_R
AA61	CCTTGCTTAGTTGCCCTTGG	POSTN_en_F
AA62	TGAGACAGCAGAGTTTACAGC	POSTN_en_R

Legend
1 = F means forward; R means reverse.
2 = F means forward; R means reverse; pr means promoter region; en means enhancer region.

Supplementary Table S5: List of antibodies used in this study.

Target	Source	Code	Dilution
CK 8	Covance	1E8-MMS-162P	1:1000
CK14	Covance	AF64-155P	1:1000
α-SMA	Abcam	ab5694	1:100
Ki67	Dako	M7240	1:50
p63	Santa Cruz Biotechnology	sc-8431	1:50
CK5	Novocastra	CK5-L-CE	1:50
CK 5/6	Dako	M723729	1:50
CK8/18	Novocastra	5D3-L-CE	1:50
Vimentin	CST	3932	1:100
PR	Biocare Medical	CM-424-A	1:50
Her2	CST	4290	1:100
ER	Novocastra	NCL-L-ER-6F11	1:50
MYC	Santa Cruz Biotechnology	sc-764	4μg/50μg chromatin
trimethyl histone H3 Lys4	Millipore	07-473	4μg/50μg chromatin
monomethyl histone H3 Lys4	Abcam	8895	4μg/50μg chromatin
acethyl histone H3 Lys27	Abcam	4729	4μg/50μg chromatin
MIZ-1	Walz, S. et al., Nature 511, 2014	10E2	80μl/50μg chromatin
β-Actin	Sigma-Aldrich	A5441	1:5000
c-Myc	Cell Signaling	5605	1:1000
Pan-AKT	Cell Signaling	2920	1:1000
P-AKT (Thr308)	Cell Signaling	2965	1:1000
GAPDH	Santa Cruz	sc-32233	1:1000
P-AKT (Ser473)	Cell Signaling	4060	1:2000
ER-α	Merck Millipore	F3-A 04-1564	1:500
