# Supplementary material for: MYC-driven epigenetic reprogramming favors the onset of tumorigenesis by inducing a stem cell-like state
Source: Nat Commun. 2018 Mar 9;9:1024. doi: 10.1038/s41467-018-03264-2 (PMC5844884; doi:10.1038/s41467-018-03264-2)
Supplement: Supplementary file 3 — Description of Additional Supplementary Files [file 41467_2018_3264_MOESM3_ESM.pdf]

Description of Additional Supplementary Files

File Name: Supplementary Data 1
Description: List of gene signatures used for GSEA analyses in the paper
